# Efficient Caoshu Character Recognition Scheme and Service Using CNN-Based Recognition Model Optimization

**DOI:** 10.3390/s20164641

**Published:** 2020-08-18

**Authors:** Boseon Hong, Bongjae Kim

**Affiliations:** 1 Department of Computer and Electronics Convergence Engineering, Sun Moon University, Asan 31460, Korea; goodcools34@gmail.com; 2 Artificial Intelligence Research Center, Korea Electronics Technology Institute, Seongnam 13488, Korea; 3 Division of Computer Science and Engineering, Sun Moon University, Asan 31460, Korea

**Keywords:** convolutional neural networks, mobile services, Caoshu recognition, model optimization, data augmentation

## Abstract

Deep learning-based artificial intelligence models are widely used in various computing fields. Especially, Convolutional Neural Network (CNN) models perform very well for image recognition and classification. In this paper, we propose an optimized CNN-based recognition model to recognize Caoshu characters. In the proposed scheme, an image pre-processing and data augmentation techniques for our Caoshu dataset were applied to optimize and enhance the CNN-based Caoshu character recognition model’s recognition performance. In the performance evaluation, Caoshu character recognition performance was compared and analyzed according to the proposed performance optimization. Based on the model validation results, the recognition accuracy was up to about 98.0% in the case of TOP-1. Based on the testing results of the optimized model, the accuracy, precision, recall, and F1 score are 88.12%, 81.84%, 84.20%, and 83.0%, respectively. Finally, we have designed and implemented a Caoshu recognition service as an Android application based on the optimized CNN based Cahosu recognition model. We have verified that the Caoshu recognition service could be performed in real-time.

## 1. Introduction

Various services are becoming intelligent with the development of artificial intelligence technologies such as deep learning [1,2,3,4,5,6,7]. Deep learning technology is widely applied in many fields such as image processing, network, security, IoT, medical, and so on. For example, the Convolutional Neural Network (CNN) model shows outstanding performance in areas such as image recognition and image classification [8,9,10,11,12,13]. CNNs are simply neural networks that use convolution operation in place of general matrix multiplication in at least one of their layers. Various services are designed and implemented based on CNN. Representative CNN models include SPP-net [14], YOLO (You Only Look Once) [15], AlexNet [16], GoogLeNet [17], ResNet [18], VGGNet [19], SSD [20], and DenseNet [21]. As various CNN-based models have been studied, recognition and classification performance is also improving.

The Caoshu is a kind of cursive characters in Chinese. Caoshu characters have a characteristic by being written in a much more abstract form, unlike ordinary Chinese characters. Besides, old documents or books with many historical values were often written in Caoshu characters. Lastly, it is a difficult problem since there are a large number of character classes [22]. Even old literature researchers are having a hard time deciphering or interpreting Caoshu based scripts. Caoshu characters can be recognized effectively using CNN-based image recognition and classification technology. Therefore, an optimized recognition model is needed to efficiently classify and recognize Caoshu characters based on a CNN model. An effective recognition service for researchers based on the Caoshu recognition model is also needed. In this paper, we propose an optimized CNN-based Caoshu character recognition model to recognize Caoshu characters. In our Caoshu recognition scheme, we use the DenseNet-201 model as a base CNN model. The DenseNet (Densely connected convolutional Networks) is a kind of Convolutional Neural Networks. It is one of the best performing CNN models. In addition, we propose an online Caoshu recognition service for old literature researchers.

We use our self-made Caoshu character dataset for training, validation, and testing of the proposed Caoshu recognition model. The Caoshu character dataset was constructed with characters that are frequently used. Our Caoshu dataset consists of a total of 527 Chinese character classes, and the total number of data is 38,878. Each class consists of an average of about 73 images. The number of the data constituting each class is unbalanced. The 527 character classes of Caoshu were digitized as a JPEG image format from actual old books for training, validating, and testing the proposed model. The original of our Caoshu dataset has a data count of 38,878. It can be considered as a relatively small dataset to train the CNN model when compared to other datasets such as the ImageNet [23] dataset and Microsoft COCO [24] dataset. We apply two data augmentation techniques to improve recognition performance with a limited dataset. The data augmentation techniques used to extend the dataset are the data scale transformation technique and the data affine transformation technique. The Caoshu dataset with data augmentation techniques consists of a total of 61,348 images.

In the performance evaluation, the *K*-fold cross-validation method was applied to evaluate the recognition performance. We used a 5-fold cross-validation scheme. The performance of Caoshu recognition was compared and analyzed according to our optimization techniques in terms of accuracy. Based on the model validation results, the recognition accuracy of our optimized Caoshu character recognition model was up to about 98.0% in the case of TOP-1. Based on the model testing results, the accuracy, precision, recall, and F1 score are 88.12%, 81.84%, 84.20%, and 83.0%, respectively.

Finally, we have implemented an online Caoshu recognition service as an Android application that is operated by the optimized CNN based Cahosu recognition model. It has the advantage of easy to use because it was implemented in the form of an Android application. Through actual implementation, it was verified that the Caoshu recognition service could be performed in real-time. Through our application, the user can select a character to be recognized and then check the recognition result. Providing services like our online Caoshu recognition can help researchers who are studying old literature.

The rest of this paper is organized as follows. In Section 2, we give an introduction to the background and related works. Section 3 presents the proposed CNN-based Caoshu character recognition model. Section 4 is a performance evaluation of the proposed Caoshu recognition model. We will explain our Caoshu recognition service in Section 5. A further discussion of experimental results is presented in Section 6. Finally, Section 7 summarizes the paper and gives concluding remarks.

## 2. Related Works

We will explain some related researches in this section. First of all, the convolutional neural network is a representative artificial neural network that shows excellent image recognition and classification performance. In general, a convolutional neural network is composed of convolution layers, activation layers, and pooling layers. At the last layer, it classifies recognition results through a fully connected layer. The convolutional neural network was used by the team that won first place in ImageNet Large Scale Visual Recognition Challenge (ILSVRC) [16,21,25,26,27]. Various services based on CNN are continuously being researched. The following are studies related to the recognition of Chinese handwriting based on CNN models.

Zhong et al. [28] proposed high performance offline handwritten Chinese character recognition based on GoogLeNet. The proposed CNN model is a streamlined version of the original GoogLeNet. To enhance the performance of GoogLeNet in terms of recognition accuracy, it employed three types of directional feature maps: the Gabor, gradient, and HoG feature maps. The modified GoogLeNet was consisted of 19 layers but involved only 7.26 million parameters. However, it is not a study using Caoshu characters.

Lee et al. [29] also proposed a ResNet-based percussive network for cursive Chinese characters recognition. The Chinese character dataset with 824 classes was used to evaluate the performance. They used 10-fold cross-validation for evaluation of accuracy and confirmed that 94.7% classification accuracy was measured. Though the dataset used for evaluation is different, our optimized Caoshu recognition model shows higher accuracy.

Xiao et al. [30] proposed a method for building fast and compact CNN model for large scale handwritten Chinese character recognition. They designed a nine-layer CNN for handwritten Chinese character recognition. They focused on fast recognition while minimizing performance degradation in terms of recognition accuracy.

Wang et al. [31] proposed a hierarchical CNN model for recognizing confusable and similar handwritten Chinese characters. The proposed hierarchical CNN model takes advantage of deep networks and traditional hierarchical methods. The proposed CNN model consists of two stages. The first stage is expected to differentiate inter-group characters. The second stage is to differentiate intra-group characters. They used 368 similar characters that are extracted from 3755 frequently used Chinese characters. Experiments have shown that similar Chinese characters can be distinguished well through the proposed technique.

Tang et al. [22] proposed a transfer learning method based on Convolutional Neural Network (CNN) for historical Chinese character recognition. They tried to overcome the problem of lacking sufficient labeled training samples via a transfer learning method. Printed Chinese character samples train a CNN model. Then, the model is enhanced by a few historical or handwritten data. They showed the proposed method is effective based on several experiments.

Melnyk et al. [32] proposed a new CNN-based method called Melnyk-Net for offline handwritten Chinese character recognition and visualization. They used a modified global weighted average pooling. The main purpose of the proposed method was to reduce the size of the trained model. The average accuracy of the proposed method was 97.61%.

As described in this section, several studies have been conducted for Chinese recognition. However, there are not many CNN-based studies of Chinese handwriting with a high degree of abstraction such as Caoshu characters. Therefore, we focus on Caoshu characters that Chinese handwriting with a high degree of abstraction in this paper. We propose an optimized Caoshu character recognition model based on CNN in this paper. In the case of Chinese, there is a problem that there are many types of letters, and the data to be used for training the recognition model is relatively small. We also applied three optimization techniques to our DenseNet-201 based CNN model to improve the accuracy of Caoshu recognition because the total number of our Caoshu dataset is relatively small. Based on the experimental results, the degree of accuracy is comparable to or better than the existing research. Furthermore, we have implemented the Caoshu recognition service as an Android application to verify the practical use of the proposed optimized Caoshu recognition model. As far as we know, there have been no studies that have designed, implemented, and verified real-time Caoshu recognition services. Therefore, our research is different from existing research. By the above reasons, it can be said that there are contributions with the differentiation from existing research.

## 3. An Optimized CNN-Based Caoshu Character Recognition Model

In this section, we present an optimized CNN-based Caoshu character recognition model. First, our optimization methods for improving recognition performance will be described. Then, we will describe the DenseNet-201 CNN model used in this paper. Table 1 shows the notations and their descriptions used in this paper.

### 3.1. Performance Optimization Techniques

#### 3.1.1. Changes in the Size of Input Image

**Figure 1:**
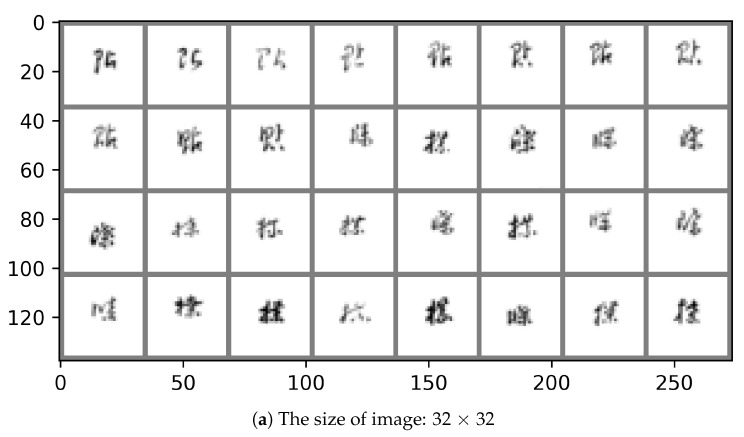

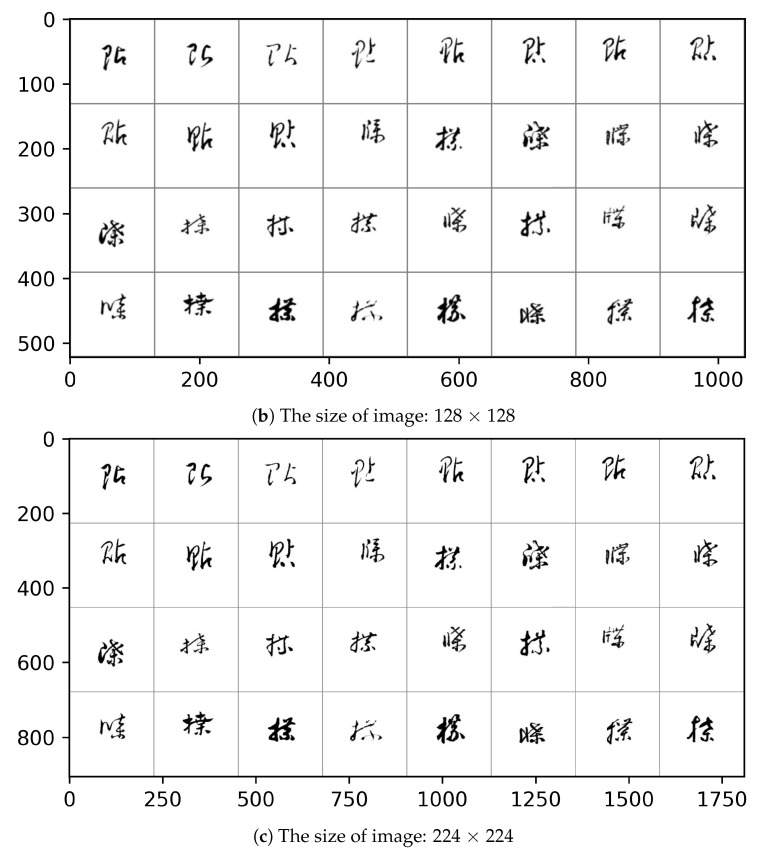
An example image according to the size of 32 × 32, 128 × 128, and 224 × 224, respectively.

The size of the input image used in the input layer of the CNN model is related to recognition performance. Therefore, the size of the CNN model’s input image for the recognition of Caoshu characters can be changed to optimize the recognition performance. In general, the recognition accuracy increases as the size of the image used in the CNN model increases. The original size of each image is 224 × 224. We downsampled to change the size of the image. Figure 1 shows an example image according to the size of 32 × 32, 128 × 128, and 224 × 224, respectively. In this paper, the image size is changed to 32 × 32, 128 × 128, and 224 × 224, and optimized to increase recognition accuracy.

#### 3.1.2. Image Binarization

We pre-processed the images in the dataset to remove noise, such as unnecessary points in the image. As a pre-processing technique, an image binarization technique was applied to remove noises of each image of our Caoshu dataset.

(1) $$D_{Binarization}\left(x,y\right)=\left\{\begin{array}{c} 255,D\left(x,y\right)\geq Decision_{TH}\\ 0,D\left(x,y\right)<Decision_{TH} \end{array}\right.$$

**Figure 2:**
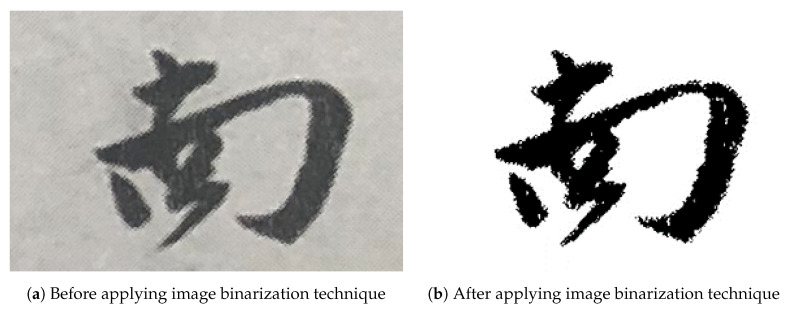
An example of image binarization technique.

The concept of our image binarization technique is expressed as Equation (1). $D_{Binarization}\left(x,y\right)$ means the binarized value of an image. (x,y) means the pixel position of an image. D(x,y) denotes the original value of an image where 0 ≤ D(x,y) ≤ 255. $Decision_{TH}$ is a threshold value for image binarization. The value of 0 indicates black, and the value of 255 indicates white. Figure 2 shows an example by the image binarization technique. In the process of image data pre-processing, the $Decision_{TH}$ value was set to 90. In short, if the value of the pixel is greater than 90, it is converted to white. Contrary, if it is less than 90, it is converted to black.

#### 3.1.3. Data Augmentation

The original of our Caoshu dataset has a data count of 38,878. It can be considered as a relatively small dataset to train the CNN model. To overcome and manage this problem, we apply two data augmentation techniques: the data scale transformation technique and the data affine transformation technique.

Equations (2) and (3) are expressions of the data augmentation techniques applied to enhance the performance in terms of recognition accuracy. α is a parameter value indicating the degree of scaling of the original image in terms of the size. Similarly, θ value is a parameter value indicating the degree of rotation of the original image. As shown in Equation (2), each (x,y) position of an image data is scaled to the position $\left(x_{Scaled},y_{Scaled}\right)$ according to the factor of α. Similarly, each (x,y) position of an image data is rotated to the position $\left(x_{Affined},y_{Affined}\right)$ according to the factor of θ. The empty space created of each image by data augmentation is treated with white as the background color.

(2) $$\left(\begin{array}{c} x_{Scaled}\\ y_{Scaled} \end{array}\right)=\left(\begin{array}{c} x\\ y \end{array}\right)\left[\begin{array}{cc} \alpha & 0\\ 0 & \alpha \end{array}\right]$$

where 0.8 < α≤ 1.2.

(3) $$\left(\begin{array}{c} x_{Affined}\\ y_{Affined} \end{array}\right)=\left(\begin{array}{c} x\\ y \end{array}\right)\left[\begin{array}{cc} \cos\theta & \sin\theta \\ -\sin\theta & \cos\theta \end{array}\right]$$

where $-15^{\circ }<\theta \leq 15^{\circ }$.

**Figure 3:**
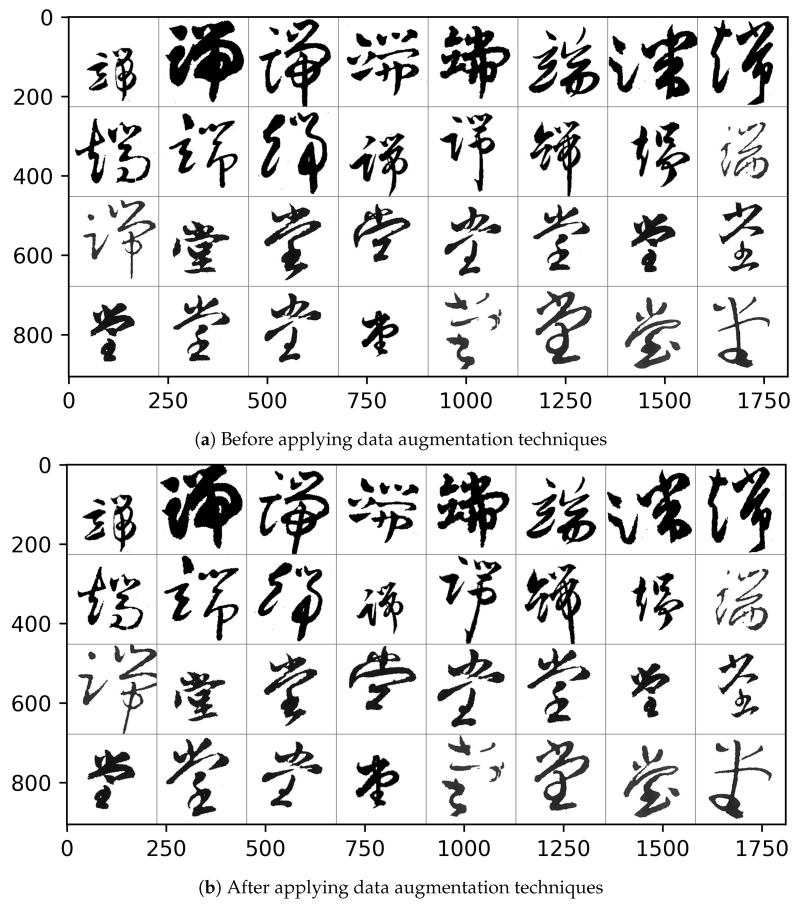
An example image of data augmentation techniques.

Figure 3 shows some examples of proposed data augmentation. As shown in Figure 3, we can confirm that the changes in terms of the size and the rotation angle of images. The α and θ are chosen as random values within the range. After applying the data augmentation techniques, the dataset has 61,348 Caoshu character images.

### 3.2. Caoshu Character Recognition Model Based on DenseNet-201 CNN Model

DenseNet (Densely connected convolutional Networks) is a kind of Convolutional Neural Networks [21]. We use a DenseNet-201 as a base CNN model for recognizing Caoshu characters. A DenseNet is a type of convolutional neural network that utilizes dense connections between each layer, through dense blocks. To preserve the feed-forward feature, each layer obtains additional inputs from all preceding layers and passes on its feature-maps to all subsequent layers in the DenseNet. The network can be thinner and compact because each layer receives feature maps from all preceding layers. The DenseNet has some advantages due to its structure. For examples, the DenseNet has a strong gradient flow. In short, the error signal can be more easily propagated to earlier layers more directly. Besides, it has a smaller size and computational efficiency when compared to previous CNN models such as ResNet. Lastly, it has more diversified features and tends to have richer patterns because each layer in DenseNet receive all preceding layers as input.

Table 2 represents the structure of modified DenseNet-201 model for cahoshu recognition. As shown in Table 2, our modified DenseNet-201 consists of one first layer, four block layers, three transition layers, and one classification layer. The first layer’s main components are 7 × 7 convolution and 3 × 3 max pooling. Each block layer’s main components are 1 × 1 convolution and 3 × 3 convolution. Each transition layer’s main components are 1 × 1 convolution and 2 × 2 average pooling. Each transition layer is used for 1/2 downsampling. In the DenseNet, the growth rate *k* is the additional number of channels for each layer. The growth rate of modified DenseNet-201 was set to 32.

**Figure 4:**
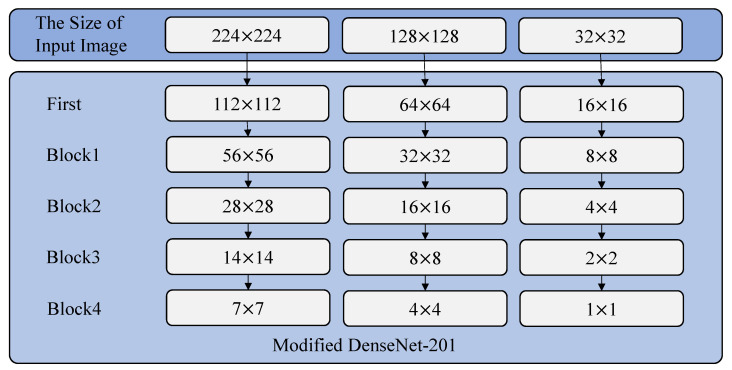
The size of the image according to each layer.

**Figure 5:**
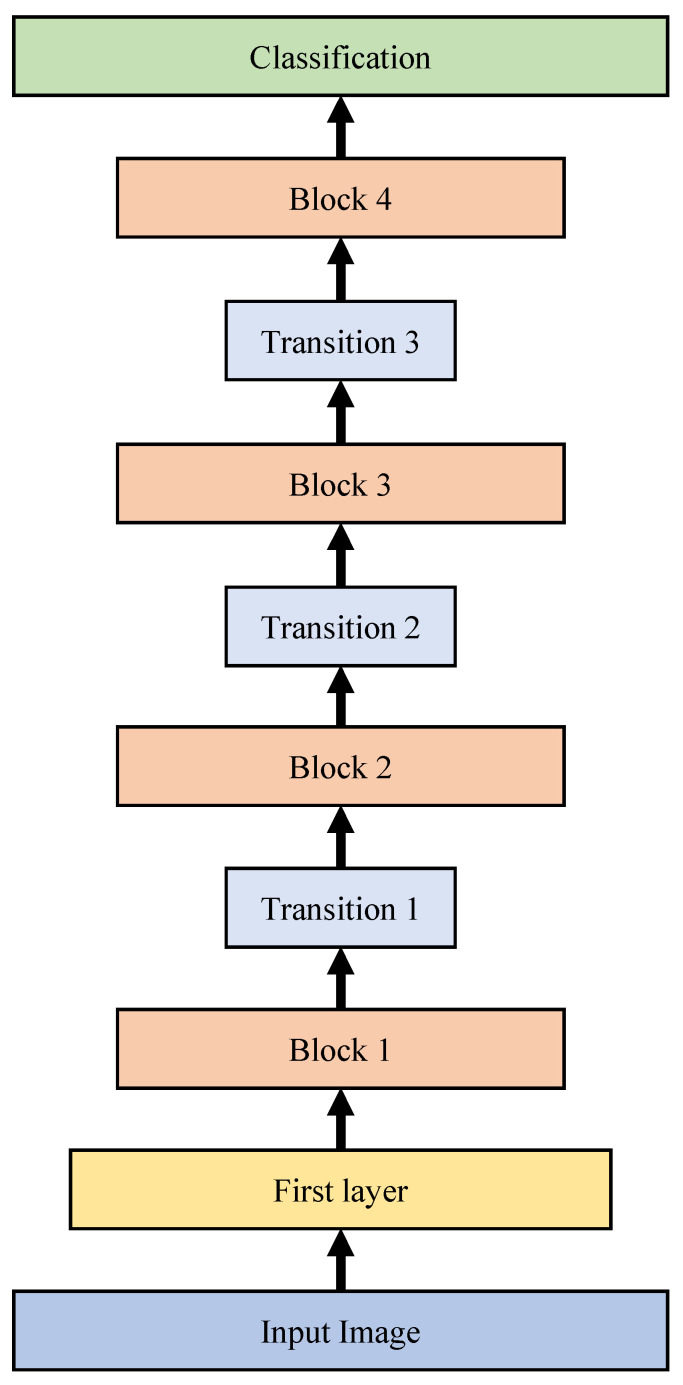
The block diagram of the modified DenseNet-201 Model.

We also modified the input layer and output layer to fit our Caoshu dataset. In the our DenseNet-201 based Caoshu recognition model, the number of output nodes of the fully connected layer was modified to 527 because the number of classes of our Caoshu dataset is 527. The size of an image for the input layer can be 32 × 32, 128 × 128, or 224 × 224 as shown in Figure 4. Figure 5 shows the block diagram of the modified DenseNet-201 model. As shown in Figure 5, the input image data passes through the first layer and finally goes to the classification layer. In this way, the recognition result can be finally obtained.

When training the proposed model, the hyper-parameters are tunned by applying a scratch learning technique. We use cross entropy as a loss function. We The stochastic gradient descent (SGD) is used for optimization. The learning rate of the model was set to 0.1, and the momentum was set to 0.9. Besides, the learning rate was set to decrease at a rate of 0.1% every 30 steps.

## 4. Performance Evaluation

### 4.1. Performance Evaluation Environment

Table 3 shows the our performance evaluation environment. The environment of the computing node used in the experiment is as follows. The CPU is Intel i7-6850K (3.6 GHz). The computing node is equipped with four NVIDIA GTX 1080Ti GPUs. The computing node has 64GB RAM. The operating system used is Ubuntu 16.04 LTS. The version of CUDA is 10.0. The deep learning framework used is PyTorch [33], and the version is 1.2. PyTorch is an open-source software for machine learning based on the Torch library. It is widely used for various applications such as computer vision and natural language processing. PyTorch is primarily developed by Facebook’s AI Research lab (FAIR). The version of Python is 3.6.

Table 4 shows the detailed information of our self-made Caoshu dataset used in the performance evaluation. Caoshu character images were extracted as a JPEG image format from actual old books. As shown in Table 4, our Caoshu dataset consists of 527 Caoshu character classes. The total number of Caoshu image data is 38,878. The original size of each image is 224 × 224. The average number of images consisting of each class is about 73.7. The minimum number of images in one class is 19. The maximum number of images in one class is 377. The standard deviation of the number of images in each class is about 51.7.

In the performance evaluation, the *K*-fold cross-validation method was applied to evaluate the recognition performance. We used a 5-fold cross-validation scheme in the performance evaluation. Recognition accuracy is analyzed and evaluated in the case of TOP-1 and TOP-5. TOP-1 means the case where the classified result matches the input data. TOP-5 means the case that the input data matches within the top five results that are sorted in order of probability.

### 4.2. Training and Validation Results of the Proposed Caoshu Recognition Model

#### 4.2.1. Training and Validation Results

**Figure 6:**
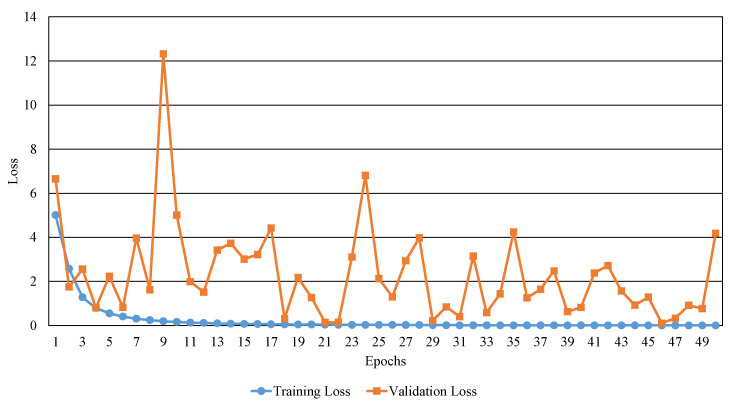
The results of training and validation losses over epochs.

**Figure 7:**
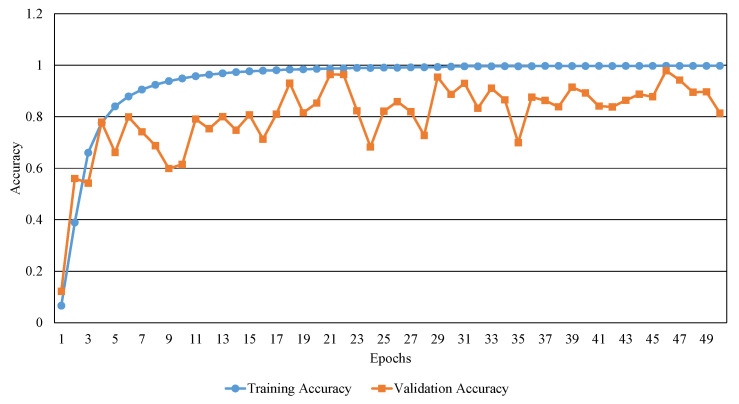
The results of training and validation accuracies over epochs.

Figure 6 and Figure 7 show the training and validation results in terms of accuracy and loss. In the case of training loss, the training loss decreases steadily as the epoch increases. In the case of validation loss, it can be confirmed that the change is relatively notable depending on each epoch, but it decreases as a whole. In the case of training accuracy, the training accuracy increases steadily as the epoch increases. The validation accuracy also tends to increase as the epoch increases. When comparing the validation loss with the training loss, the value of validation loss is relatively large than that of the training. This can be analyzed that the trained model is overfitted to training data. The overfitting problem is due to the size of our Caoshu data set used is relatively small and the number of the data constituting each class is unbalanced.

#### 4.2.2. According to the Size of Input Image

**Figure 8:**
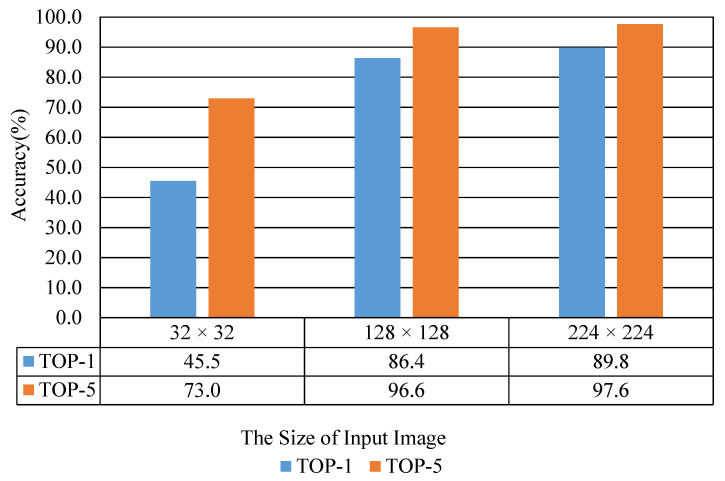
Recognition performance results according to the size of the input image.

Figure 8 shows the recognition performance results according to the size of the input image. These results are a case of using a model trained with a dataset where data augmentation was not applied. As shown in Figure 8, the performance in terms of recognition accuracy gradually increases as the size of the input image increases. As shown in Figure 8, in the case of TOP-1, the validation accuracy of the model is 89.8% when the size of the input image is 224 × 224. The validation accuracy of the model is 45.5% when the input image’s size is 32 × 32. At this time, the difference in recognition accuracy is about 44.3%. The performance difference is relatively large.

Table 5 shows the results of the inference time according to the size of input image. As shown in Table 5, there is little difference in the time required for recognition. In the case of 32 × 32, the time required for recognition is about 30.7 ms. In the case of 128 × 128, the time required for recognition is about 31.6 ms. The inference time is about 31.0 ms in the case of 224 × 224. Therefore, if we consider both conditions, it is much better to use a high-resolution input image in the input layer of the model.

#### 4.2.3. According to Data Augmentation

**Figure 9:**
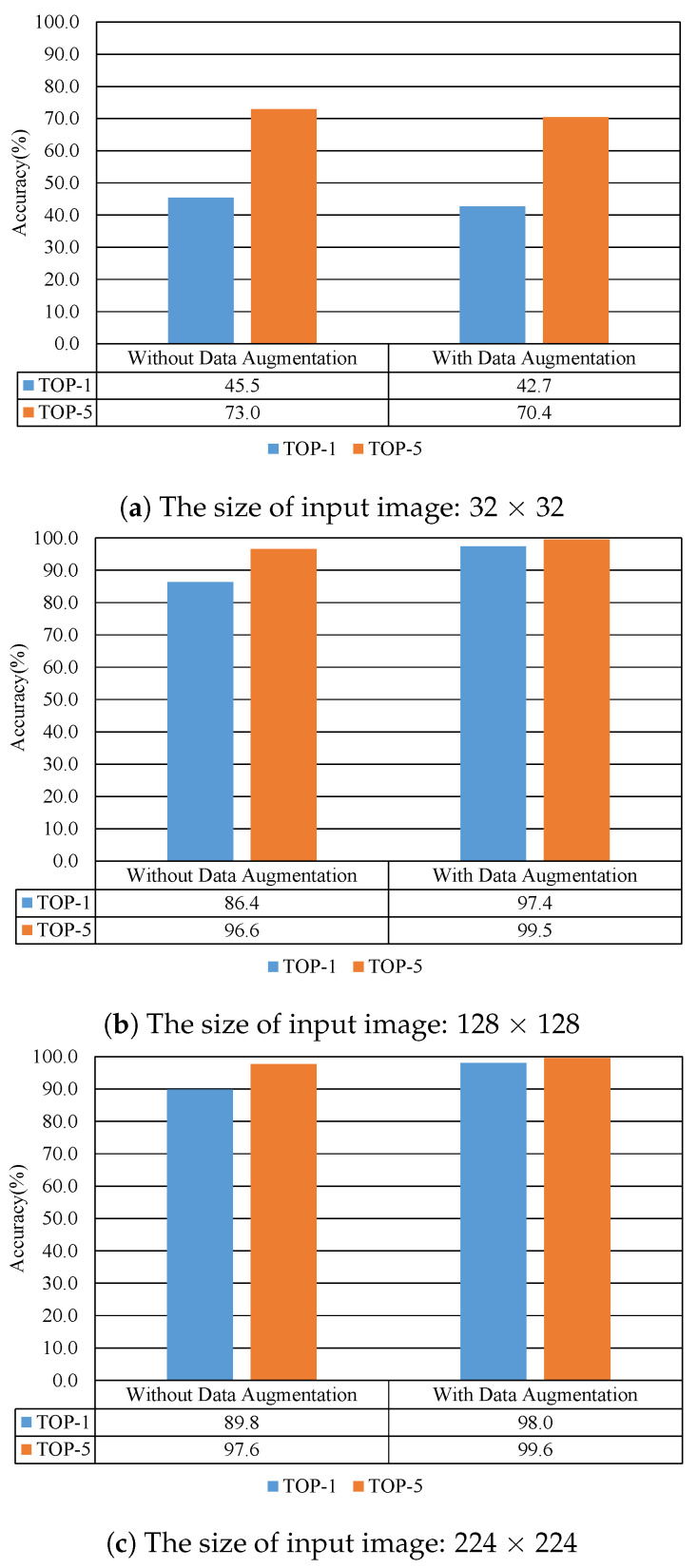
Recognition performance results according to data augmentation.

Figure 9 shows the recognition performance results according to data augmentation. As shown in Figure 9, the performance in terms of recognition accuracy increases when data augmentation techniques are applied to the dataset.

When the input image size was 224 × 224, the accuracy was increased by the data augmentation technique as follows. In the case of TOP-1, the accuracy increased by about 10.0%, and in the case of TOP-5, it increased by 2.9%. In conclusion, the accuracies of TOP-1 and TOP-5 are about 98.0% and 99.6%, respectively. Based on the results, it shows good validation results in terms of the accuracy.

However, when the input image’s size is 32 × 32, the recognition accuracy is reduced. In the case of TOP-1, the accuracy decreased by about 2.8%, and in the case of TOP-5, it decreased by 2.6%. This result means that if the input image’s size is too small, the data augmentation technique does not help to improve the accuracy.

### 4.3. Testing Results of the Trained Caoshu Recognition Model

We have tested the performance of the trained and optimized Caoshu recognition model. We used a test dataset that is not used for training and validating the Caoshu recognition model. We can estimate the performance of the proposed Caoshu recognition model in the real situation through this model testing.

Table 6 shows the testing results of the proposed Caoshu recognition model in terms of accuracy, precision, recall, and F1 score. In case of multi classes recognition tasks, the accuracy, precision, recall, and F1 score can be calculated by Equations (4)–(7), respectively. We assume that *n* is the number of classes. $Y_{i}$ is the ground truth label assignment of the *i*th class. $x_{i}$ is the *i*th class. $h(x_{i})$ is the predicted labels for the *i*th class. The higher value of accuracy, precision, recall, and F1 score, the better means the better the performance. The accuracy, precision, recall, and F1 score are 88.12%, 81.84%, 84.20%, and 83.0%, respectively. Model testing results also show relatively high performance.

(4) $$Accuracy=\frac{1}{n}\sum_{i=1}^{n}\frac{\left|Y_{i}\cap h\left(x_{i}\right)\right|}{\left|Y_{i}\cup h\left(x_{i}\right)\right|}$$

(5) $$Precision=\frac{1}{n}\sum_{i=1}^{n}\frac{\left|Y_{i}\cap h\left(x_{i}\right)\right|}{\left|h(x_{i})\right|}$$

(6) $$Recall=\frac{1}{n}\sum_{i=1}^{n}\frac{\left|Y_{i}\cap h\left(x_{i}\right)\right|}{\left|Y_{i}\right|}$$

(7) $$F1-Score=\frac{1}{n}\sum_{i=1}^{n}2\times \frac{\left|Y_{i}\cap h\left(x_{i}\right)\right|}{\left|Y_{i}\right|+\left|h(x_{i})\right|}$$

## 5. Online Caoshu Recognition Service Application

### 5.1. Service Flow of the Proposed Online Caoshu Recognition System

**Figure 10:**
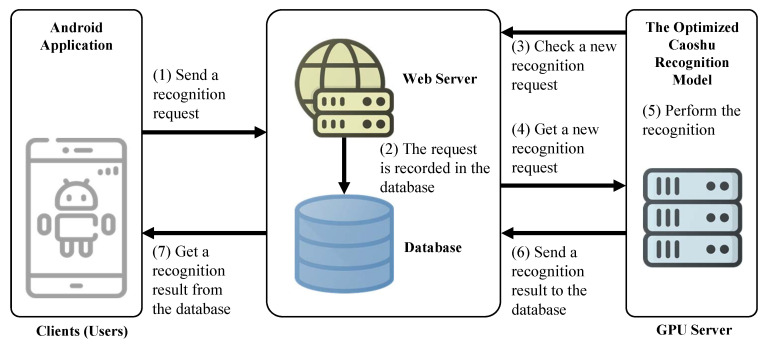
The service flow of the proposed online Caoshu recognition system.

Figure 10 shows the service flow of the proposed online Caoshu recognition system. As shown in Figure 10, our online Caoshu recognition system consists of three major components: the Android application, the Web server with a database, and the GPU server with the optimized Caoshu recognition model. The GPU server used is identical to the specifications used in the performance evaluation. Apache is used as the Web server. The version of Apache is 2.4.28. We used the MariaDB as our database system. The version of MariaDB is 10.1.38. PHP was used as the server-side language. The version of PHP is 7.1.27.

The proposed service proceeds in the following sequences as shown in Figure 10. (1) The user sends a recognition request to the web server through the Android application via RESTFul APIs. (2) The recognition request is recorded in the database. (3) In the case of the GPU server, it checks the database to get a new recognition request. (4) If there is a new request for recognition, the GPU server gets a new recognition request from the database. (5) It processes the recognition request by using the optimized Caoshu recognition model. (6) Then, the GPU server delivers the recognition results back to the database. (7) Finally, the user can check the recognition result in the Android application.

### 5.2. Implementation Results

**Figure 11:**
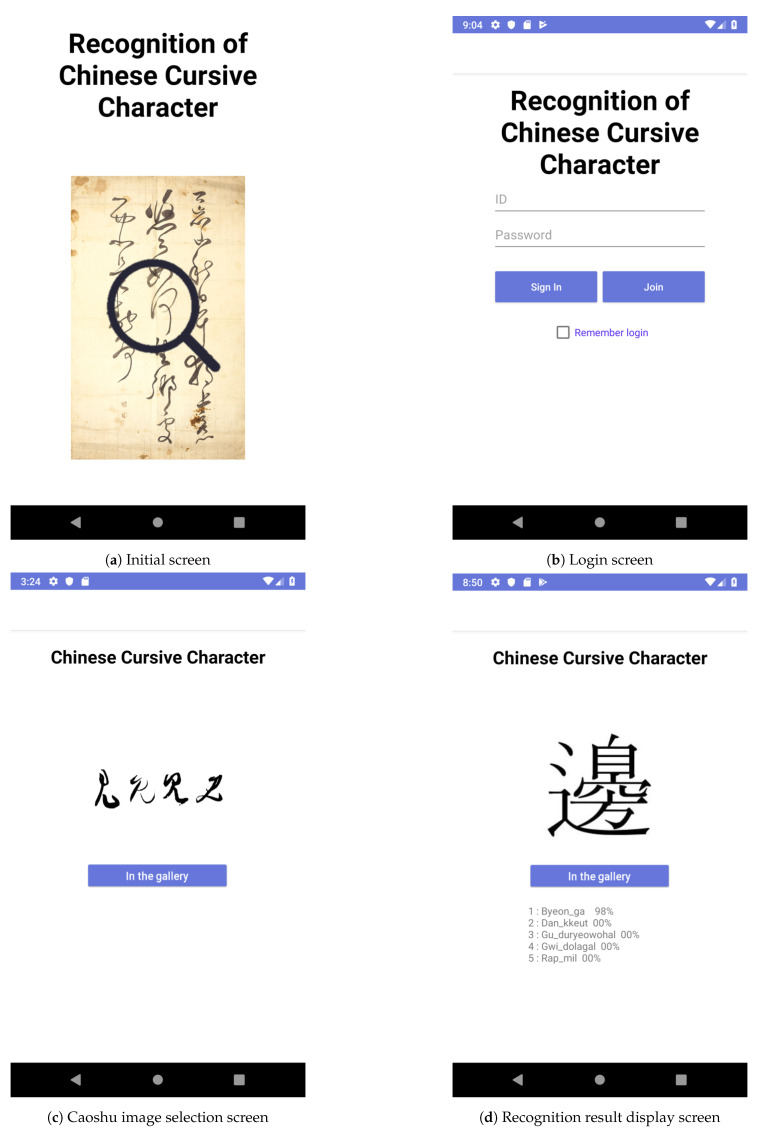
Some Examples of the implemented Caoshu recognition application.

As shown in Figure 11, we have designed and implemented an online Caoshu recognition service as an Android application. Table 7 shows the main functionalities of the implemented online Caoshu recognition service. The developed application is optimized for Android Pie 9.0. Figure 11 shows some examples of the implemented Caoshu recognition service. Figure 11a is the initial screen of our implemented application. The user can log in to the system and use our online Caoshu recognition service, as shown in Figure 11b. Then, by selecting the *In the gallery* button, the user can choose a Caoshu character to be recognized by selecting a photo existing on the mobile phone, as shown in Figure 11c. The photo selected is binarized by our binarization technique, as described in Section 3. The pre-processed image data is transmitted to our GPU server on which the trained Caoshu recognition model is operated. The recognition result is transmitted back to the user. Finally, as shown in Figure 11d, final recognition results are displayed in traditional Chinese. When showing the recognition results, five recognition results are displayed in order of inference probability. The Caoshu recognition service has been developed in the form of an Android app, so its ease of use is the most significant advantage.

## 6. Discussions

We showed that the size of the input image should be increased to increase the performance in terms of accuracy through the experimental results. We confirmed that the Caoshu recognition performance can be improved using the proposed data augmentation technique. Exceptionally, when the input layer of the Caoshu recognition model has a size of 32 × 32, the performance of the trained Caoshu recognition model with our data augmentation techniques was slightly degraded. It is analyzed that the size of the input image is too small to extract features for effective classification. Furthermore, this is due to that each Caoshu character becomes more ambiguous by the data augmentation techniques when the input data is too small. Therefore, it can be seen that the data augmentation technique should be applied when the size of the input data of the input layer is relatively large.

Our Caoshu dataset used in this paper has a relatively small dataset when compared to other datasets such as the ImageNet [23] dataset and Microsoft COCO [24] dataset. Besides, it is an unbalanced dataset with a different number of images for each class of our Caoshu dataset. With these problems, our optimized Caoshu recognition model showed a tendency to overfit the training data. In the future, this problem needs to be solved, and we think that reinforcing the dataset can be one of the solutions.

We have verified that the Caoshu recognition service could be performed in real-time based on our online Caoshu recognition service. However, there is a limitation that only one Caoshu character can be recognized at a time. There is also a limitation that the number of recognizable Caoshu characters is limited to 527 classes due to our Caoshu dataset. We have a plan to extract Caoshu image data from actual old documents or books to expand the recognizable Caoshu characters in the future.

## 7. Conclusions

Various services are becoming intelligent based on artificial intelligence technology, such as deep learning and machine learning. These intelligent services are showing better performance than before. The CNN model shows excellent recognition performance in the field of image recognition and classification. In this paper, we proposed an optimized CNN-based Caoshu character recognition model to recognize Caoshu characters. It was confirmed that the performance in terms of recognition accuracy was improved by optimizing the CNN model for Caoshu character recognition. According to the experimental results by 5-fold cross-validation, the proposed DenseNet-201 based optimized Caoshu character recognition model showed about 98.0% recognition accuracy in the case of TOP-1. Based on the testing results of the optimized model, the accuracy, precision, recall, and F1 score were measured by about 88.12%, 81.84%, 84.20%, and 83.0%, respectively. It is a fairly high level of performance although we used a small Caoshu dataset. Finally, we have implemented the Caoshu recognition service as an Android application. Based on the implementation of the Caoshu recognition service, we have verified that the practical use of the proposed optimized Caoshu recognition model. It can be used as a very effective tool for old literature researchers.

## Figures and Tables

**Table 1:** Notations and their descriptions.

Notations	Descriptions
(x,y)	The pixel position of an image
$Decision_{TH}$	A threshold value for image binarization
D(x,y)	The original value of the position (x,y) of an image, where 0 ≤ D(x,y) ≤ 255
$D_{Binarization}\left(x,y\right)$	The binarized value of the position (x,y) of an image, where $D_{Binarization}\left(x,y\right)$ is 0 or 255
α	A parameter value indicating the degree of scaling of an original image in terms of the size
θ	A parameter value indicating the degree of rotation of an original image
$\left(x_{Scaled},y_{Scaled}\right)$	The scaled position of the position (x,y) of an image
$\left(x_{Affined},y_{Affined}\right)$	The rotated position of the position (x,y) of an image

**Table 2:** The structure of modified DenseNet-201 model for Caoshu recognition.

Layers	Core Layers	# of Core Layers	Remarks
First	7 × 7 Conv. and 3 × 3 Max. Pool.	-	
Block 1	1 × 1 Conv. and 3 × 3 Conv.	6	
Transition 1	1 × 1 Conv. and 2 × 2 Avg. Pool.	-	1/2 Downsampling
Block 2	1 × 1 Conv. and 3 × 3 Conv.	12	
Transition 2	1 × 1 Conv. and 2 × 2 Avg. Pool.	-	1/2 Downsampling
Block 3	1 × 1 Conv. and 3 × 3 Conv.	48	
Transition 3	1 × 1 Conv. and 2 × 2 Avg. Pool.	-	1/2 Downsampling
Block 4	1 × 1 Conv. and 3 × 3 Conv.	32	
Classification	527 Fully-connected, Softmax		

**Table 3:** Performance evaluation environment.

Feature	Description
CPU	Intel i7-6850K (3.6 GHz)
GPU	NVIDIA GTX 1080Ti 11 GB × 4
RAM	64 GB
OS	Ubuntu 16.04 LTS
CUDA Version	CUDA 10.0
Deep Learning Framework	PyTorch 1.2
Python Version	Python 3.6

**Table 4:** The detailed information of our self-made Caoshu dataset.

Features	Descriptions
The Number of Classes	527
The Total number of Caoshu Image data	38,878
The Original Size of Each Image	224 × 224
The Average Number of Images Consisting of Each Class	73.7
The Minimum Number of Images in One Class	19
The Maximum Number of Images in One Class	377
The Standard Deviation of The number of Images in Each Class	51.7

**Table 5:** Inference time of the proposed Caoshu recognition model.

The Size of Input Image	Inference Time (Unit: ms)
32 × 32	30.7
128 × 128	31.6
224 × 224	31.0

**Table 6:** Performance results of the proposed Caoshu recognition model.

Feature	Result
Accuracy	88.12%
Precision	81.84%
Recall	84.20%
F1 Score	83.00%

**Table 7:** Main functionalities of the implemented online Caoshu recognition service.

Features	Descriptions
Supporting Multi Users	Multiple users can use the service at the same time through membership registration and login functions.
Real-time Recognition of Caoshu Characters	It is possible to recognize one Caoshu character at a time.
Number of Recognizable Caoshu Characters	A total of 527 Caoshu characters are supported.
